# Quality by Design for the Development and Analysis of Enhanced In-Situ Forming Vesicles for the Improvement of the Bioavailability of Fexofenadine HCl In Vitro and In Vivo

**DOI:** 10.3390/pharmaceutics12050409

**Published:** 2020-04-29

**Authors:** Ali M. Nasr, Mona K. Qushawy, Mahmoud M. Elkhoudary, Aya Y. Gawish, Sameh S. Elhady, Shady A. Swidan

**Affiliations:** 1 Department of Pharmaceutics, Faculty of Pharmacy, Port Said University, Port Said 42526, Egypt; 2 Department of Pharmaceutics, Faculty of Pharmacy, Sinai University, Alarish, North Sinai 45511, Egypt; 3 Department of Pharmaceutics, Faculty of Pharmacy, University of Tabuk, Tabuk 71491, Saudi Arabia; 4 Department of Pharmaceutical Chemistry, Faculty of Pharmacy, Horus University-Egypt, New Damietta 34518, Egypt; melkhodary@horus.edu.eg; 5 Department of Pharmacology & Toxicology, Faculty of Pharmacy, MTI University, Cairo 11571, Egypt; gawishaya@gmail.com; 6 Department of Natural Products and Alternative Medicine, Faculty of Pharmacy, King Abdulaziz University, Jeddah 21589, Saudi Arabia; ssahmed@kau.edu.sa; 7 Department of Pharmaceutics, Faculty of Pharmacy, The British University in Egypt, El-Sherouk city, Cairo 11837, Egypt; 8 The Center for Drug Research and Development (CDRD), Faculty of Pharmacy, The British University in Egypt, El-Sherouk City, Cairo 11837, Egypt

**Keywords:** enhanced in-situ formed vesicles, absorption enhancers, fexofenadine, allergic rhinitis, experimental design, central composite design

## Abstract

Drug absorption from the gastrointestinal tract (GIT) is one of the major problems affecting the bioavailability of orally absorbed drugs. This work aims to enhance Fexofenadine HCl oral bioavailability in vivo, the drug used for allergic rhinitis. In this study, novel spray-dried lactose-based enhanced in situ forming vesicles were prepared using different absorption enhancer by the slurry method. Full factorial design was used to obtain an optimized formulation, while central composite design was used to develop economic, environment-friendly analysis method of Fexofenadine HCl in plasma of rabbits. The optimized formulation containing Capryol 90 as absorption enhancer has a mean particle size 202.6 ± 3.9 nm and zeta potential −31.6 ± 0.9 mV. It achieved high entrapment efficiency of the drug 73.7 ± 1.7% and rapid Q3h release reaches 71.5 ± 2.7%. The design-optimized HPLC assay method in rabbit plasma could separate Fexofenadine HCl from endogenous plasma compounds in less than 3.7 min. The pharmacokinetic study and the pharmacological effect of the fexofenadine-loaded optimized formulation showed a significant increase in blood concentration and significantly higher activity against compound 48/80 induced systemic anaphylaxis-like reactions in mice. Therefore, enhanced in situ forming vesicles were effective nanocarriers for the entrapment and delivery of Fexofenadine HCl.

## 1. Introduction

Oral route is the preferred route of administration for the majority of the patients especially, for long periods of treatment, as it is non-invasive, flexible, and mostly of lower cost compared to other routes. This can explain that the oral route for drug delivery alone represents 52% of the overall market share, representing the dominant route of the overall pharmaceutical market [1]. In spite of the aforementioned advantages, poor bioavailability remains a major obstacle for most drugs administered orally. Poor solubility and permeability, low absorption rate in the GIT environment, and first pass effect are the major obstacles form which orally administered drugs suffered [2]. In order to develop oral dosage forms that can overcome these drawbacks, several factors should be tested to find the optimum formulations as well as the most suitable analysis method. Quality by design approach (QbD) proved successfulness in the understanding of formulation and analysis factors and their interaction effects by a desired set of experiments. Quality by design is a broad term that refers to the achievement of certain predictable quality with desired and predetermined specifications [3]. The traditional approach in the development and optimization of multifactor experiments was studying the influence of the corresponding factors by changing one variable at a time (OVAT) approach, whilst keeping the others constant. The OVAT methods were proved inefficient because the global optimum might not be found, and the concluded optimal conditions might depend on the starting conditions [4]. On the other hand, a multivariate approach varies several factors simultaneously. An experimental design is an experimental set-up that allows studying simultaneously a number of factors in a predefined number of experiments. Roughly, experimental designs can be divided into screening designs (e.g., full factorial, fractional factorial, and Plackett–Burman designs), response surface designs and mixture designs. Full factorial designs (FFD) and Response surface experimental designs (RSD) are amongst types of designs used to find the optimal levels of the most important factors affecting the experiment, which are selected based on experimenter experience or screening designs [5]. Parallel to the QbD advances, the tremendous growth of basic and clinical nanomedicine studies and the development of novel nanoparticles suitable for oral administration also allowed a huge improvement of oral bioavailability for poorly bioavailable drugs [6]. For poorly absorbed drugs, nanotechnology-based vesicular systems showed attractive properties, such as higher absorption rate, good biocompatibility and targetability. The major problems of these nanovesicles are stability problems especially in lipid-based vesicles such as fusion and aggregation [7]. In addition, the aqueous nature of these nanovesicle vehicles can hinder their effective usage. In situ forming nanovesicles is a novel approach that involve the preparation of dry, free-flowing surfactant-coated provesicular nano drug carriers, which upon hydration and gentle agitation in water before the oral administration form nanovesicles that facilitate the absorption of the included drugs [8]. As they formulated from surfactants, they can overcome the stability drawbacks associated with lipid nanoparticles such as liposomes, nanoemulsions and solid lipid nanoparticles. In situ formed nanovesicles have advantage over the navovesicles in aqueous media which suffers from leakage aggregation and fusion as well as poor chemical stability [9]. The inclusion of intestinal absorption enhancers to the provesicular system can enhance the absorption of poorly absorbed drugs. Enhanced in situ forming vesicles (EIFV) are novel provesicular systems that contain some absorption enhancers such as Capryol 90, Maisine CC, Labrafil M 1944, Labrasol, Chremophor, etc., in addition to the commonly used nonionic surfactants forming the vesicles. These oils are reported to enhance both membrane permeability and intestinal absorption of number of poorly absorbed drugs [10,11,12]. Different studies showed also that they can improve the bioavailability of absorbed compounds by facilitating transcellular and paracellular absorption [13]. EIFV are the optimum candidate delivery system for poorly soluble and poorly permeable drugs such as Fexofenadine HCl (FEX). FEX is an orally administered non-sedating antihistamine. It is the active metabolite of terfenadine, a well-known and effective H1 receptor antagonist. It is characterized by low oral bioavailability approximately 33% in humans [14], this is due to its low intestinal permeability and also because the intestinal P-gp limits its oral absorption as it is a substrate for P-glycoprotein [15]. It is an antihistamine providing rapid, long-acting, and highly selective peripheral H1 receptor antagonist activity. FEX cannot pass through the blood–brain barrier, so it has non-sedating antihistaminic action [16]. It is one of the drugs of choice for the treatment of allergic rhinitis, which is a symptomatic inflammatory disorder of the nose induced after allergen exposure by an immunoglobulin E(IgE)-mediated inflammation. The main symptoms of this allergy are sneezing, nasal obstruction, and mucous discharge [17]. In this study FEX was formulated in spray-dried lactose-based EIFV containing 3 different absorptions enhancers of different HLB values: Maisine CC, Capryol 90, and Labrafil M 1944. To our knowledge, the use of such absorption enhancers for improvement of bioavailability of drugs in provesicular systems is not yet investigated. The aim of this study is to benefit from the merits of experimental design in optimizing the preparation of FEX EIFV powder and for the development of suitable analysis method of FEX in rabbits’ plasma. It aims also to evaluate different absorption enhancers for the improvement of FEX delivery. To achieve these aims, different formulation variables were studied and the characteristics of formed EIFV will be evaluated in vitro and all variables were optimized. The optimized EIFV formulation were evaluated in vivo using an experimental design optimized and validated HPLC method using the widely available UV detection then a pharmacokinetic study and pharmacological effect studies were conducted to evaluate the effect of the novel EIFV in vivo.

## 2. Materials and Methods

### 2.1. Materials

FEX and spray-dried lactose were generously supplied as gifts from Medical Union Pharmaceuticals (MUP). Cholesterol (Chol) was purchased from Panreac Quimica SA, Barceolna, Spain. Span 40 (Sorbitan monopalmitate) and Span 60 (Sorbitan monostearate) were purchased from Oxford Laboratory Chemicals, India. Capryol 90 (propylene glycol monocaprylate), Maisin CC (glyceryl monolinoleate) and Labrafil M 1944 (oleoyl poloxyl 6 glyceride), were kindly gifted from Gattofosse, France. Compound 48/80 )the condensation product of *N*-methyl p-methoxyphenethylamine and formaldehyde) was purchased from Sigma Aldrich Sigma Chemical Co. St. Louis, MO, USA. Methanol and Chloroform (analytical grade) were purchased from Fisons, England. Acetonitrile and methanol (HPLC grade) were purchased from Fisher Scientific Ltd. All other chemicals used were of analytical grade with no further modifications. Deionized water (Milli-Q) was used.

### 2.2. Experimental Design

#### 2.2.1. Full Factorial Design (FFD) for FEX EIFV Powder Optimization

Based on extensive studies on blank spray-dried lactose-based provesicles done by our group and published elsewhere, it was found that both span 60 and span 40 are best nonionic surfactants forming surfactant-based provesicles. The best surfactant to Chol ratios were 1:1 and 2:1 respectively [18]. The FEX-loaded EIFV powders were prepared according to FFD (2^2^.4^1^) using Design-Expert 11 software (Stat-Ease, Inc., Minneapolis, MN, USA). The design has three independent factors to be studied, the type of surfactant used (X1), the type of absorption enhancer added (X2) and the surfactant to Chol ratio (X3) (Table 1). The second factor was of four categories enhancer free, and three different enhancers with varying HLB values. While particle size (Y1), zeta potential (Y2), EE% (Y3) and total amount of FEX released after 3 h (Q3 h) (Y4) were selected as responses.

#### 2.2.2 Central Composite Design (CCD) for HPLC Assay Optimization of FEX in Plasma

A rotatable CCD (RCCD) was used. In this type of design, the star points are equal to ±(2k)1/4 (α = 1.68). The information is equally generated from all directions, i.e., the variance of the estimated responses is the same at all points on a sphere centered at the origin. Six center point replications were done to consider the experimental errors. Then, the 20 experiments (N = 8 + 6 + 6) were done in random order. After careful trials and study of the factors affecting chromatographic separation, the chosen factors were pH (X1), temperature (X2), and flow rate (X3). The selected responses were retention time of FEX (Y1), retention time of tinidazole (Y2), and peak area of FEX (Y3). 

Surface plots were developed using the fitted quadratic polynomial equation and were used to locate the points of maximum response for each analyte in the considered domain. The optimal conditions were obtained by choosing the best optimum value for each chromatographic response.

### 2.3. Preparation of FEX EIFV Powder

The dry EIFV powders were prepared using the slurry method as mentioned by Gurappu et al., with slight modification [19]. Three absorption enhancers of different HLB were used. The composition and ratios of all components of the prepared formulae are shown in Table 1. In brief, 120 mg FEX and total weight of 1 gof the lipid mixture composed of, surfactant, and Chol with or without absorption enhancer were dissolved in 10 mL of chloroform methanol solvent mixture of ratio 7:3 respectively in a round bottom flask. After complete solubility, equal amount of spray-dried lactose was added to the mixture forming a slurry. The solvent mixture was then evaporated using Heidolph rotary evaporator (P/N Hei-AP Precision ML/G3, Schwabach, Germany) under pressure of 600 mmHg at a temperature of 45 ± 1 °C and speed of 60 rpm until complete dryness. The obtained dry powder forming thin film was further rotated in rotary evaporator to remove all traces of the organic solvents. Then the FEX EIHV powder was further dried overnight in desiccators at room temperature to obtain dry, free-flowing product [20]. The prepared formulations were stored in a tightly closed container for further investigations.

### 2.4. Micromeritic Properties of the Prepared FEX EIFV Powders

The micromeritic properties of the prepared FEX EIFV powders containing free-flowing spray-dried lactose as a carrier were evaluated through the measurement of the angle of repose, Hausner’s ratio and compressibility index (Carr’s index). The angle of repose was measured using fixed funnel method [21]. Carr’s index and Hausner’s ratio were calculated by measuring the bulk and tapped density of the provesicular powders according to the following equations [22]:

(1) $$\mathrm{Carr}’s\;\mathrm{index}=\frac{\rho t-\rho b}{\rho t}\times 100$$

(2) $$\mathrm{Hausner}\;\mathrm{ratio}=\frac{\rho t}{\rho b}$$

where *ρb* and *ρt* are the bulk density and tapped density respectively.

### 2.5. Formation of the Nanovesicles from FEX-Loaded Provesicular Powders 

FEX-loaded provesicular dry free-flowing powders were transformed into nanovesicles by hydration with 10 mL distilled water warmed to at 37 °C with gentle agitation using Thermolyne Vortex Mixer (Thermo Scientific, Maxi-Mix II, 120V, 50/60Hz, TX, USA) for 2 min.

### 2.6. Particle Size Analysis and Surface Charge Determination

Particle size (PS), polydispersibility index (PDI), and zeta potential (ZP) of the formed nanovesicles were determined by photon correlation spectroscopy (PCS) using the Malvern Zetasizer (Nano ZS, Malvern Instruments Ltd., Malvern, United Kingdom). The samples were appropriately diluted with distilled water to have a suitable scattering intensity. All measurements were done at room temperature (25 °C) and in triplicates. The mean values and SD were calculated. 

### 2.7. Determination of FEX Entrapment Efficiency in the Formed Nanovesicles

The percentage of FEX entrapped in the nanovesicles formed after the hydration of the EIFV powders was calculated by indirect method. Briefly, accurately weighed EIFV powder was hydrated with phosphate buffer pH 7.4 and vortexed for 2 min. The formed vesicular dispersion was centrifuged using cooling centrifuge at 15,000 rpm at 4 °C. Aliquot of 500 μL of the supernatant was withdrawn and the amount of unentrapped FEX was determined at 221 nm using UV spectrophotometer (Jenway 6305 spectrophotometer, Staffordshire, UK). The EE% was calculated using the following equation:

(3) $$\mathrm{EE}\;\%=\frac{\mathrm{total}\;\mathrm{amount}\;\mathrm{of}\;\mathrm{FEX}-\mathrm{amount}\;\mathrm{of}\;\mathrm{FEX}\;\mathrm{in}\;\mathrm{the}\;\mathrm{supernatent}}{\mathrm{total}\;\mathrm{amount}\;\mathrm{of}\;\mathrm{FEX}}\times 100$$

This experiment was done in triplicate and both mean and SD were measured.

### 2.8. In Vitro Dissolution study of FEX Release from the Prepared EIFV

The in vitro dissolution study of FEX EIFV powders was done as described by Veerareddy et al., with slight modifications [23]. The dissolution study was done using paddle apparatus (USP type II) (VISION^®^ G2 Classic 6™, Hanson, CA, USA). The dissolution medium was 900 mL of phosphate buffer (pH 7.4). The experiment performed at a temperature of 37 ± 0.5 °C with paddle speed set at 25 rpm throughout the experiment. The solubility of FEX in the dissolution medium was examined in order to ensure that the sink condition was maintained. At predefined time intervals up to 4 h, an aliquot of 3 mL was withdrawn, and the samples were filtered through 0.45 µm membrane filter (Millipore, CA, USA) and the cumulative drug release was determined spectrophotometrically at 221 nm. After each sample withdrawal, equal volume of preheated dissolution medium was placed in the vessels to compensate the withdrawn volume.

### 2.9. Morphology and Surface Characteristics of the Optimized FEX EIFV Powder and the Formed Nanovesicles

#### 2.9.1. Scanning Electron Microscopy

The surface characteristics of the optimized formulation of FEX EIFV powder were studied by scanning electron microscopy (SEM). Small amount of the dry powder of the optimized formulation was coated with approximately 15 nm gold (SPI-Module Sputter Coater). The golden-coated sample then has been scanned by analytical scanning electron microscope (JSEM-6360LA, JEOL, Tokyo, Japan) under vacuum conditions at 15 kV acceleration voltage at room temperature. 

#### 2.9.2. Transmission Electron Microscopy

The optimized formulation was examined using transmission electron microscopy (TEM) by negative staining technique (JTEM-1010, JEOL, Tokyo, Japan). One drop of the nanovesicular dispersion was added onto a carbon-coated copper grid coating, and then the excess liquid droplets were removed by a filter paper. After 5 min, one drop of uranylacetate solution (2% *w*/*v*) was then dropped onto the grids. The sample then was air-dried at room temperature and the examination was done at 74 kV. The obtained TEM image was analyzed for size distribution by the software Nano Measurer 1.2.5 (Fudan University, Shanghai, China). From the obtained data, d10, d50, and d90 were calculated.

### 2.10. Thermal Analysis

The physical nature and crystallinity of FEX in the optimized formulation was evaluated using differential scanning calorimetry (DSC) analysis. This thermal analysis was done by differential scanning calorimeter (DSC 6000; Perkin Elmer, Waltham, MA, USA). Pure FEX powder, span 40, Chol, and the physical mixture of these powders in addition to the optimized EIFV formulation were added individually to aluminum seal pan, then covered with aluminum cover. All samples were scanned over the temperature range from 25 to 200 °C at 10 °C/min under nitrogen purge at 30 mL/min. The reference material used in the analysis is pure Indium (In).

### 2.11. Pharmacokinetic Study of FEX in Rabbits

#### 2.11.1. The Design of the Pharmacokinetic Study

The in vivo pharmacokinetic study was done according to Gundogdu et al. [16], on twelve rabbits (the weight is 2–2.5 kg and provided by the animal laboratory, Faculty of Pharmacy, The British University in Egypt). The rabbits were randomly divided into two groups—six rabbits in each group—and all rabbits were housed at room temperature (25 ± 0.5 °C) and light-controlled room of two cycles 12 h light and 12 h dark. On the day before the experiment, all rabbits were fastened overnight for 12 h. They had free access to water and the remained conscious throughout the whole experiment. One group were administered powdered crushed Telfast^®^ tablet and the other administered the powder of the optimized formula, both in the same dose of 6 mg/kg body weight. Two milliliters blood samples were withdrawn from the ear vein at predetermined time intervals (0, 0.5, 1, 2, 3, 4, 5, 6, 7, 8 h). Blood samples were immediately centrifuged at 10,000 rpm for 15 min and the obtained plasma samples were stored at −20 °C until they were assayed by HPLC procedure mentioned below. The in vivo pharmacokinetic study was carried out according to the guidelines approved by the ethics committee of Faculty of Pharmacy, The British University in Egypt, approval number Ex-1904 (approval date: 8 August 2019).

#### 2.11.2. Drug Assay in Plasma

##### Instrumentation

The HPLC (Hitachi LaChrome Elite, Tokyo, Japan) instrument was equipped with a model series L-2000 organizer box, L-2300 column oven, L-2130 pump with built-in degasser, Rheodyne 7725i injector with a 20 μL loop and a L-2455 photo diode array detector (DAD), separation and quantitation were made on a 250 × 4.6 mm (i.d.), 5µm Inertsil ODS-2 column (Gl Sciences, Tokyo, Japan). UV detection was performed under scan mode (in the range of 200–350 nm with a 1 nm distance) and multiwavelength overlay chromatograms for quantitative analysis. The HPLC was operated by EZchrom Elite version 3.3.2 SP1 by Agilent. 

##### Chromatographic Parameters

An HPLC method was developed and optimized using the previously mentioned CCD. The best composition of the mobile phase through isocratic elution was prepared by using 20 mM phosphate buffer and adjusted with 1 M HCl to a pH of (3.07 ± 0.01) as mobile phase A—acetonitrile as mobile phase B (80:20 *v*/*v*). The flow rate was maintained at 0.93 mL min^−1^. The mobile phase was filtered through a 0.45-µm disposable filter (Milliopore Milford, MA). All determinations were performed at 40 °C. The injection volume was 20 μL. Quantitation was achieved with UV detection at 215 nm for FEX and 319 nm of tinidazole (TNZ) as internal standard (IS) based on peak area.

##### Preparation of Standard and Calibration Solutions

Stock standard solutions were prepared by dissolving 25 mg of FEX in 50 mL methanol. The dissolution was made with the help of ultrasonic bath for about 15 min. The calibration standard working solutions were prepared by dilution of the stock standard solution with the initial mobile phase composition to reach the concentration range of 10–1000 µg mL^−1^. Triplicate 20 μL injections were made for each drug concentration level and chromatographed under the conditions described above. All stock and working standard solutions were kept away from light to avoid photodegradation.

##### Sample Preparation

The calibration solutions were prepared by transfer of 50 µL of each FEX working solutions, 50 µL of TNZ (IS), and 400 µL of blank plasma to set of centrifugation tubes then vortex mixed for 1 min and centrifuged at 3000 rpm for 5 min. 20 µL of the clear and filtered supernatant were then injected into HPLC. Extraction of rabbit plasma samples was done by adding 50 µL of IS to 450 µL of plasma then they were treated as calibration solutions.

##### Assay Validation

The developed and optimized HPLC method was validated according to the guidelines for bioanalytical method validation set by the Food and Drug Administration (FDA) [24]. The method was validated for selectivity, linearity, accuracy, precision, matrix effect, and stability of FEX in spiked plasma samples. Selectivity was tested by analyzing rabbit blank plasma samples from six different rabbits at the lower limit of quantification (LLOQ) samples. Linearity was assessed by plotting calibration curves in human urine between peak area ratio of the analytes to the IS solutions to the analyte concentration. The curves were fitted by a linear weighted (1/x^2^) least-square regression method.

#### 2.11.3. Pharmacokinetic Analysis

The pharmacokinetic parameters of FEX was calculated for each rabbit in both groups using pharmacokinetic software (PK function for Microsoft Excel, Pharsight Corporation, Sunnyvale, CA, USA). Data from the plasma concentration versus time curve within 8 h after oral intake of both powdered market product and optimized EIFV powder formulation groups were analyzed using non-compartmental analysis. Peak plasma concentration (C_max_), time taken to reach plasma concentration (t_max_), half-life time (t_1/2_), the area under the curve (AUC_0–8 h_), and the mean residence time (MRT) were calculated. The relative oral bioavailability of the optimized EIFV formulation and the marketed product was calculated using the following Equation (4):

(4) $$\mathrm{Relative}\;\mathrm{bioavailability}=\frac{AUC\;\mathrm{of}\;\mathrm{the}\;\mathrm{optimized}\;\mathrm{formula}}{AUC\;\mathrm{of}\;\mathrm{the}\;\mathrm{marketed}\;\mathrm{powder}}\times 100$$

### 2.12. Pharmacological Evaluation of the FEX in the Optimized Formula

#### 2.12.1. Effect of FEX EIFV-Optimized Powder on Compound 48/80-Induced Systemic Anaphylaxis-Like Reactions in Mice 

Five groups each of 8 male albino mice were used in this experiment. Mice were housed 4 per cage and were maintained at room temperature (25 ± 0.5 °C). All experiments were performed in compliance with the guidelines approved by the ethics committee of Faculty of Pharmacy, The British University in Egypt (approval number Ex-1904). The mice in the first group were administered phosphate buffer saline orally (negative control group), while the second group were administered phosphate buffer saline 1 hour prior to an intraperitoneal injection of 8 mg/kg of the mast cell degranulator compound 48/80 which induces systemic anaphylaxis-like reactions in mice (positive control group). In the other groups, the pure drug powder, the marketed product crushed to be powder, and the optimized formulation powder respectively were dispersed in phosphate buffer saline and administered orally 1 h prior to compound 48/80 administration. The mortality was monitored for 1 h after induction of anaphylactic shock. After the mortality test, blood was obtained from the heart of each mouse by cardiac puncture [25,26]. 

#### 2.12.2. Preparation of Plasma and Histamine Level Determination 

The obtained blood samples were centrifuged at 400 g for 10 min in a cooling centrifuge at 4 °C. The plasma was withdrawn, and the histamine content was measured by the o-phthalaldehyde spectrofluorometric procedure as mentioned by Shore et al. [27]. The fluorescent intensity was measured at 438 nm in a spectrofluorometer.

### 2.13. Statistical Analysis

Analysis of variance (ANOVA) was performed in CCD, it was also used for the evaluation of a significant difference between groups in the pharmacological effect evaluation test. Unpaired Student t-test was used for the analysis of the data obtained from the pharmacokinetic study for untransformed data for the pharmacokinetic parameters C_max_, t_1/2,_ AUC_0–8 h_. The statistical calculations were done using the software SPSS 11.0 (SPSS Inc., Chicago, USA). A statistically significant difference was considered at *p*-value < 0.05.

## 3. Results and Discussion

### 3.1. Experimental Design

#### 3.1.1. Full Factorial Design (FFD) for FEX EIFV Powder Optimization

##### The Effect of Formulation Variables on the PS of the Formed FEX EIFV

All FEX EIFV powders were successfully prepared by the slurry methods. The formulations 1–4 contains no absorption enhancers, while the other formulations contain 3 different enhancers with different HLB and viscosity values. Formulations 5–8 contain Maisine CC of HLB value equals 1. Formulations 9–12 contain Capryol 90, which has higher HLB value equal to 5. It is of an intermediate HLB value between the vesicles forming nonionic surfactants span 40 (HLB: 6.7) and span 60 (HLB: 4.7). Formulations 13–16 contain Labrafil M 1944, it has the highest HLB value compared to other used enhancers of value equals 9.

**Figure 1:**
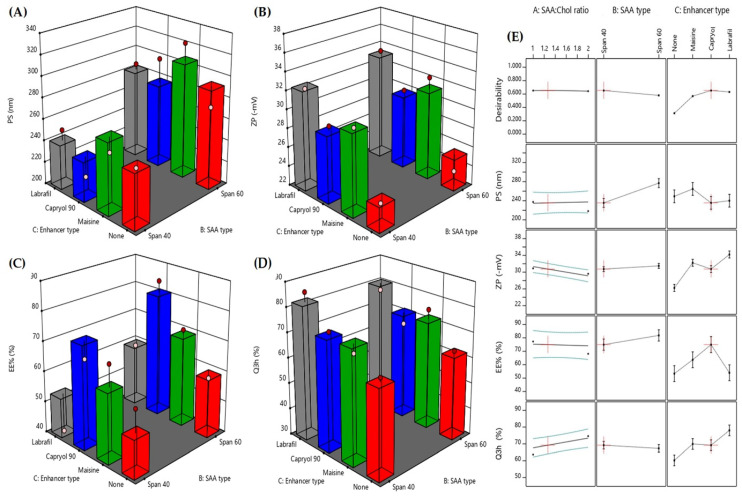
Response 3D plots for the effect of the studied formulation factors on the obtained responses (**A**)–(**D**) and the desirability (**E**). * (SAA) is surfactant.

The vesicles formed from the formulations of this experimental design had a PS ranging from 218.1 to 323.9 nm as shown in Table 2 As seen from the optimization graphs shown in Figure 1A and 1E, the main variable affecting the particle size is the type of surfactant. Using span 40 resulted in smaller vesicles compared to the formulations contain span 60. This might be due to the shorter chain of span 40. Different studies suggest that surfactant with longer alkyl chains generally give larger vesicles [28,29]. This can also explain the smaller PS obtained in all formulations that contains Capryol 90, Labrafil M and enhancer free formulations compared to those contains 18 C atoms chain Maisine CC. As seen from Table 2, the PDI of all vesicles obtained by the hydration of the prepared FEX EIFV ranged from 0.267 to 0.389. This data and the charts obtained from the Malvern zetasizer indicate that the particles are homogenous and monodisperse. All formulae had PDI less than 0.4 which indicates acceptable particle size distribution range as mentioned by Izham et al. [30]. It was noticed that the two formulations with PDI less than 0.3 contain span 40 as the surfactant and the surfactant: Chol ratio was 2:1. Both formulations showed also small particle size values.

##### The Effect of Formulation Variables on the ZP of the Obtained FEX EIFV

Zeta potential is an important parameter that its value can be related to the stability of colloidal dispersions containing nanovesicles. It is the difference in potential between the surface of tightly bound layer (shear plane) and electroneutral region of the solution. Zeta potential indicates the degree of repulsion between similarly charged, adjacent particles in dispersion. For particles that are small enough—such as nanoparticles—a high value of zeta potential will confer stability which causes a dispersion to resist agglomeration. When zeta potential is low, attraction exceeds repulsion and the dispersion will break and/or flocculate. So, colloids with high zeta potential either negative or positive are electrically stabilized [31]. As seen from Table 2, all formulations have negative zeta potential with values range from −23.5 to −36.5 which indicates the formation of stable nanovesicular dispersion after the hydration of highly stable FEX EIFV dispersion. From Figure 1B,E, the variable that most affect the zeta potential is the type of absorption enhancer. Addition of Labrafil M1944 which is the most polar absorption enhancer resulted in increase in the zeta potential, while the enhancer free formulations showed the lowest zeta potential values. This was in complete agreement with Kamboj et al., who found that the effect of HLB values of the emulsifying absorption enhancers on zeta potential could be explained in terms of surface energy, which tends to increase with increase in HLB values towards the hydrophilicity. Increase in surface energy of the vesicles leads to increase the values of zeta potential towards negative [32]. It is also clear that the addition of all types of enhancers had a positive effect on the surface charge of the formed nanovesicles compared to formulations with no absorption enhancers. Both Capryol 90 and Maisine CC gave highly negative ZP values close to that obtained by of Labrafil M1944.

##### The Effect of Formulation Variables on the EE of the Formed FEX EIFV

The addition of absorption enhancers to FEX EIFV formulations was not only for the enhancement of the drug absorption and permeation through the GIT wall, but also, they assumed to have an impact on the encapsulation of the drug within the formed vesicles after hydration. That is why the enhancers used have medium to low HLB values. Higher HLB values could have negative effect on the drug encapsulation within the formed nanovesicles. As shown in Table 2, the EE% values varied greatly and ranged from 33.34 and 77.60 %. Higher variation in EE% of drugs in the nanovesicles was found also in the study of Kamboj and coworkers where the EE% in different span-based niosomes varied from 37 to 96% [32]. This can be attributed to different factors affecting the EE% of the FEX such as the HLB of the surfactant forming the vesicles and the absorption enhancers and the enhancer type. Regarding the vesicle forming nonionic surfactant, and as seen in Figure 1C, our findings were in total agreement with the literature as different studies showed that the lower HLB values and longer alkyl chain length of the surfactants resulted in higher entrapment of hydrophobic drugs [19,33]. Similar results were obtained by El-Alim et al., who achieved the highest EE% of the lipophilic drug benzocaine into nonionic surfactant-based vesicles formulated with the lowest HLB values. They concluded that the lower the HLB of the surfactant the higher the EE%. Considering the lipophilic nature of the drug and its low water solubility the surfactant having HLB 4.7 could be beneficial to achieve higher EE% values compared to surfactant having HLB value of 6.7 [34]. Chol plays effective role in increasing the EE% of the drug within the membrane of the vesicles [35]. Higher percentage of Chol was found to achieve higher EE% of FEX. Surfactant:Chol ratio 1:1 was favorable for the drug to accommodate within the vesicles compared to 2:1 ratio. The same finding was mentioned by different studies, which found that surfactant:Chol ratio 1:1 is the best ratio for achieving the maximum EE% of the lipophilic drugs [19,32]. The role of Chol. in increasing the entrapment of the drug is discussed in details by Kumar et al., who stated that the effect of Chol in lipid bilayers of niosomes is to modulate their mechanical strength, cohesion and their permeability to the surrounding aqueous phase [19]. By the addition of Chol, the fluidity of nanovesicles vesicles is changed considerably as cholesterol imparts rigidity to vesicles, which is very important under high stress conditions [36]. The interaction of Chol with Span 60 in the bilayer of the vesicles is due to hydrogen bonding [19].

Formulations containing Capryol 90 showed higher EE%, while enhancer free formulations and Labrafil M1944 formulations showed the least FEX entrapment within the formed nanovesicles. The HLB of Capryol 90 is a medium value between the HLB of span 60 and span 40, which are the best surfactant for the formulation of surfactant-based nanovesicles such as niosomes and achieved the highest EE% among all other used surfactant as concluded by many studies. The optimum HLB found by Ahmed et al., for the entrapment of Piroxicam was (6) [37], while Yoshioka and coworker found that the highest EE% of 5(6)-Carboxyflourescein achieved using span 60 and 40 compared to low EE% when Span 20, span 80 and span 85 were used for noisome preparation [38].

##### The Effect of Formulation Variables on the in Vitro Dissolution Profile of the Formed FEX EIFV

**Figure 2:**
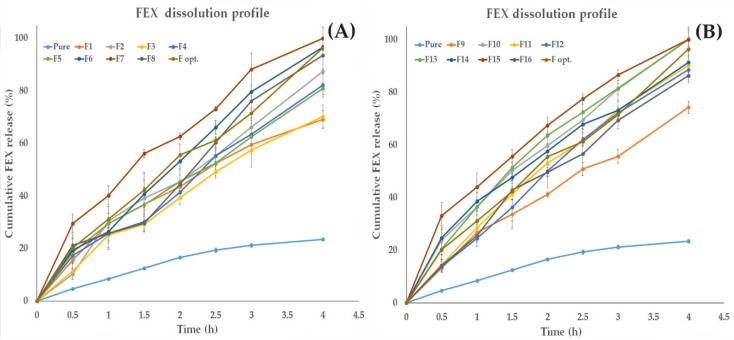
In vitro dissolution profile of all prepared FEX EIFV compared to FEX powder (**A**) formulations 1–8 (**B**) formulations 9–16.

Figure 2 shows the in vitro release of the drug in the dissolution medium (pH 7.4) for all prepared FEX EIFV compared to FEX powder. As seen from the Figure 2, after 4 h, the formulation of FEX into nanovesicles containing surfactant enhanced the release of the drug compared to the pure powder regardless of the composition and the enhancer types. Table 2 shows the drug amount released after 3 h. The cumulative percentage of the released drug after 3 h ranged from 55.68% to 88.19%. From the design analysis (Figure 1D,E), each factor that participate in increasing the polarity of the vesicles was effective in accelerating the drug release compared to formulations contains highly hydrophilic environment. The presence of Chol in a high ratio (surfactant:Chol ratio 1:1) resulted in decreasing the release of the drug compared to the formulation in which the Chol is of lower ratio. Nasr et al., who investigated the effect of the Chol ratio on the in vitro release of Aceclofenac found the formulations with higher percentage of Chol showed the slowest release compared to other formulations [33]. This might be explained by the fact that the presence of Chol within the bilayers of the vesicles at a temperature above the transition temperature (Tc) modulates the vesicles’ membrane fluidity by restricting the movement of the relatively mobile hydrocarbon chains, thus reducing bilayer permeability [39], and condenses the packing of the phospholipids in the bilayers, thus decreasing the efflux of the encapsulated drug [40], resulting in higher drug retention within the vesicles [33]. Span 40-based nanovesicles formulations showed faster drug release compared to the corresponding span 60-based formulations. The higher HLB value of the surfactant allows better solubilization of the drug in the aqueous medium. Many studies found that the use of span 60 showed slower release compared to span 40, which is attributed to the alkyl chain length of the surfactant [33]. Another suggested explanation is mentioned by Attia et al., who found that the use of span 60 niosomes resulted in slower release of Acyclovir compared to niosomal formulations containing span 20, span 80, and span 85. They attributed this effect to the fact that at 25 °C, the molecules of span 60 are in the ordered gel state, but those of other spans are in the disordered liquid crystalline state [41]. It was noticed that the addition of absorption enhancers improved the FEX release compared to the enhancer free formulations. It is also clear that the higher HLB of the enhancer resulted in faster dissolution of FEX. 

##### Optimization

The aim of the optimization process of drug formulations was to determine the levels of the studied factors required to produce a product with highest quality possible. FEX EIFV were optimized for the responses Y1 (PS), Y2 (ZP), Y3 (EE%), Y4 (Q3h). The goal of the optimization design is to maximize ZP, EE%, and Q3 and to minimize PS. The optimum values of the variables were obtained by numerical analysis using the Design-Expert^®^ 11 software and based on the criterion of desirability [42]. The levels of the formulation factors and the predicted responses of the formulation suggested from the optimization design which had the highest desirability value (0.650) is shown in Table 3. In order to confirm the validity of the optimization design and process, the optimal FEX EIFV formulation with the predicted levels of the independent variables was prepared and characterized. The observed responses of the optimum formulation are presented in Table 3. The PDI of the optimized formulation is 0.393 ± 0.020. The obtained results showed that there is high similarity between the observed and predicted responses of the optimal formulation. This makes the optimized formulation a promising nanocarrier for oral delivery of FEX. Hence, further investigations of the optimized formulation were done.

#### 3.1.2. Central Composite Design (CCD) for HPLC Assay Optimization of FEX in Plasma

**Figure 3:**
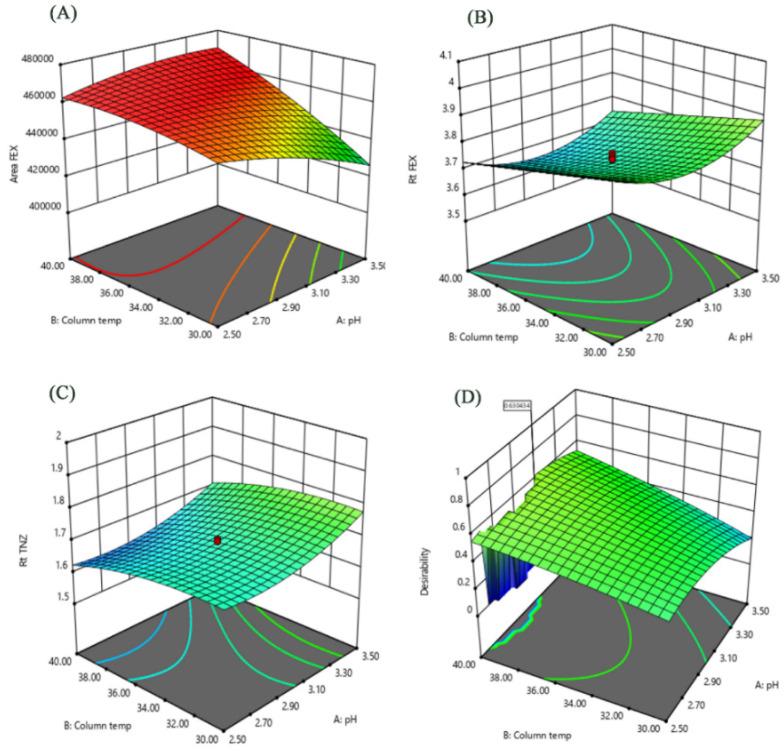
Calculated response surfaces show the interaction of pH and Column temperature on (**A**) Fexofenadine HCl peak area, (**B**) Fexofenadine HCl retention time. (**C**) Tinidazole (IS) retention time and (**D**) desirability function results.

The results obtained by the statistical analysis (ANOVA) of the studied factors and effects are given in Table 4. The insignificant terms were eliminated from the model through backward elimination process. An independent factor had a significant effect on a given response when it had a *p*-value < 0.05. The results showed that column temperature (factor B) had the most important effect on peak area FEX (response Y3). Whilst flow rate (factor C) had the most significant effects on retention times of FEX (response Y1) and TNZ (response Y2). Quadratic terms showed relatively lower significant effects. A^2^ showed a significant effect on retention times of FEX and TNZ, while C^2^ showed a significant effect on area FEX. Also, factor interactions had significant effects, especially on area. Perturbation plots are presented to show the effect of each factor on a specific response with all other factors held constant at reference point. Curvature or slope steepness indicates sensitiveness to a specific factor. Increasing levels of B resulted in an increase in peak area FEX and a decrease in both retention times of FEX and TNZ. On the other hand, decreasing levels of both factors (pH and flow rate) resulted in an increase in peak area FEX and a decrease in both retention times of FEX and TNZ. Curvature in most perturbation plots indicates quadratic significance. Response surfaces of the interaction effects of pH and column temperature are illustrated in Figure 3. It shows that peak area FEX vary in a nearly linear descending pattern, whereas retention times of FEX and TNZ exhibit a linear ascending one. Based on the results of Figure 3, optimization of the separation conditions could be concluded. The criteria for that optimization will be based on maximum peak area FEX, lowest retention time within the range of 3 to 4 min for FEX peak, and a retention time within 1.6 to 1.8 min for TNZ peak to ensure good separation from endogenous peaks. 

**Figure 4:**
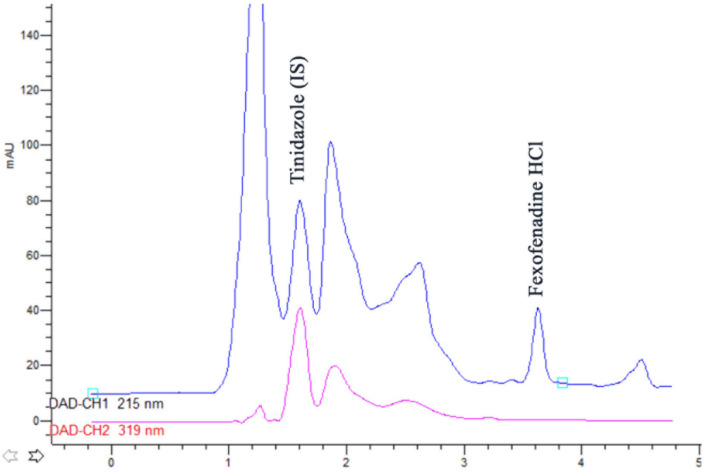
HPLC chromatogram for the analysis of rabbit plasma sample 1 h. after the administration of the optimized formulation loaded with FEX (3.61 min) showing the separation from Tinidazole (IS) and endogenous plasma compounds.

The analysis of response surfaces concludes that there is the need to make a compromise between the optimums of each response separately. Graphical and mathematical methods were used for global optimization of the separation conditions. The inspection of the desirability ramps shows that high desirability values were obtained by increasing column temperature and decreasing flow rate, while a compromise should be done for optimum pH conditions. Therefore, the following conditions were found optimum: pH 3.07, column temperature 40 °C and mobile phase flow rate 0.93 mL/min. These conditions had a desirability value of 0.63. The chromatogram obtained by these conditions is shown in Figure 4. Investigation of model predictability the difference between predicted and observed (actual) values were assessed by calculating the prediction error and a small difference between predicted and observed response values was obtained.

### 3.2. Micromeritic Properties of the Prepared FEX EIFV Powders

The angle of repose values for all prepared formulations ranged from 23.45° to 41.08°. The Carr’s index and Hausner ratio of the most flowable formulation (F2) were 14.7 and 1.172 respectively and for the least flowable formulation (F8) were 25.36 and 1.333 respectively. It was found that formulations (F 5–8) had the lowest flowability and compressibility values and they were also a little bit difficult to handle, relatively of low yield and not easily converted into dry powder after preparation. This might be due to the oily nature of Maisine CC and its high viscosity value compared to other enhancers. The viscosity of Maisine CC is 120 mPa.s at 20° C which is relatively high viscosity compare to the Capryol 90 and Labrafil M1944 which have viscosity 20 and 85 mPa.s respectively. It is clear also that the presence of Chol in high ratio (1:1) to the surfactants affected the flowability to a small extent. Formulations that do not contain absorption enhancer oil had better flowability and compressibility properties especially those with the lower ratio of Chol (Table 5). 

### 3.3. Morphological Evaluation of the Optimized FEX EIFV Powder and the Formed Vesicles

**Figure 5:**
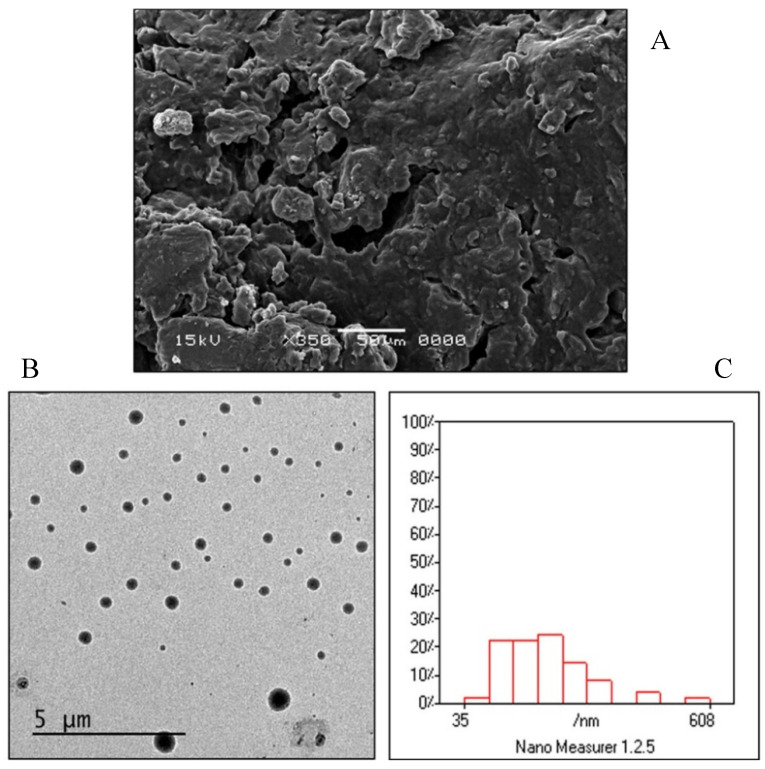
Morphological analysis of the FEX EIFV powder and the formed vesicles of the optimized formulation. (**A**) Scanning electron micrograph. (**B**) Transmission electron micrograph. (**C**) Analysis of the TEM image by Nano measurer^®^ software.

SEM images (Figure 5A) showed that the EIFV powder of the optimized formulation is nonporous with smooth surface. This might be due to the coating of the carrier surface with the surfactant [43]. The SEM image also showed the absence of any crystalline particles of the drug on the surface of the carrier.

TEM was performed to study vesicle morphology that revealed that the formed vesicles from optimized FEX EIFV formulation were well-identified perfectly spherical in shape, and they exist in discrete dispersed entities as shown in Figure 5B. The size analysis of the TEM image by Nano Measurer software (Version 1.2, Shanghai, China) showed that the vesicle size and distribution were comparable to those obtained by the DLS measurement. The vesicles were homogenous in size with narrow size distribution (Figure 5C). From the analysis of the software for the TEM image, the d10 value was 83.96 nm, d50 was 185.41, while the d90 was 322.90. The Span value which equals (d90–d10)/d50 was 1.28 which indicates narrow size distribution of the particles.

### 3.4. Thermal Analysis

**Figure 6:**
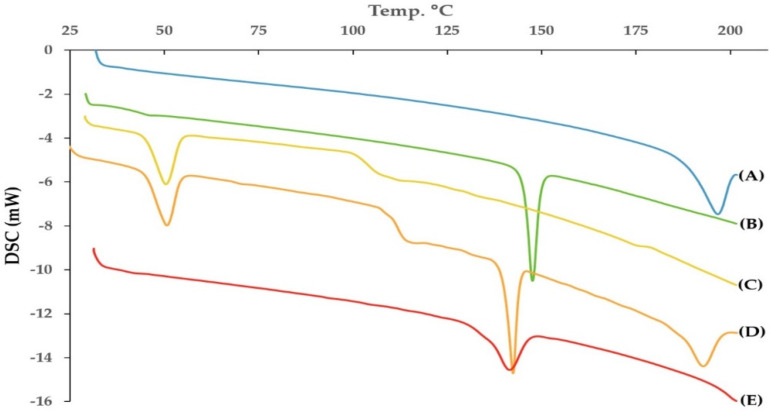
DSC thermogram of (**A**) pure Fexofenadine HCl, (**B**) Cholesterol, (**C**) span 40, (**D**) Physical mixture, (**E**) Optimized formulation powder.

DSC is an important technique used to elucidate any possible interactions of the active ingredient with other ingredients such as the carrier or the vesicle forming substances. It also proves either the drug is of amorphous or crystalline nature within the prepared formulation [44]. The DSC thermograms of FEX, Chol, and span 40 are shown in Figure 6. As seen from the Figure 6, The FEX showed melting endothermic peak at 196.72 °C. Kumar et al., mentioned that the pure FEX powder has been reported to have a melting point of 193–199 °C [45]. The enthalpy fusion (delta H) was 59.03 J/g The DSC curve of FEX powder revealed a typical behavior of crystalline anhydrous substance. Chol and span 40 showed sharp endothermic peaks at 147.58 C and 50.41 C respectively, with an enthalpy of fusion of 50.56 J/g and 46.02 J/g respectively. The DSC thermogram of the physical mixture of the active and the vesicle forming substances showed the endothermic peaks of the FEX, span 40 and Chol with slight shift of the peaks to be 191.01 °C, 50.73 °C and 143.16 °C respectively. This indicated that the drug remained in crystalline form in the physical mixture. The thermogram of the prepared optimized FEX EIFV formulation revealed that the drug did not show any melting endotherm while the peak specific for Chol appeared with slight broadening and shifting of the melting endotherm indicating interaction of Chol with other formulation components. The absence of a conspicuous peak over the range of 190–200 °C in the optimized FEX EIFV powder might be an indication of the transformation of the native crystalline form of the drug to molecular or amorphous state when dispersed in the surfactant/Chol mixture [14,46]. The transformation of FEX from crystalline to amorphous or molecularly dispersed form is beneficial for enhancing the dissolution as an amorphous form of the drug does not require energy to break up the crystalline lattice [47].

### 3.5. Pharmacokinetic Study of FEX in Rabbits

**Figure 7:**
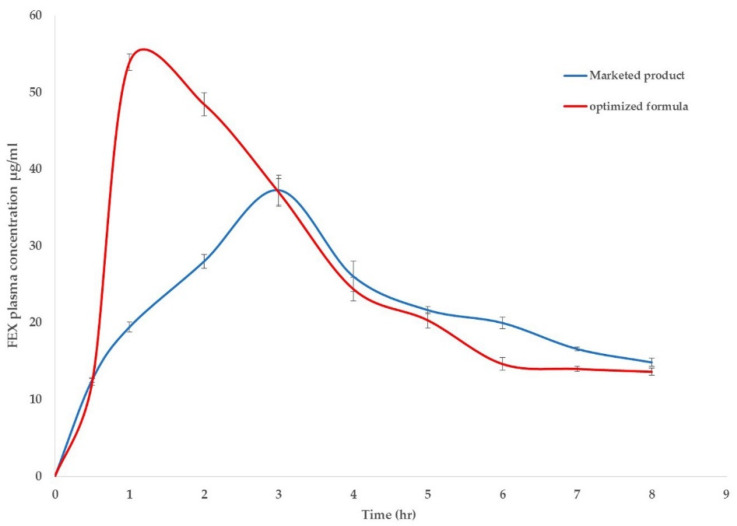
Blood concentration–time profile of FEX after oral administration of the optimized FEX EIFV powder and Telfast^®^ powdered tablet to rabbit (mean ± SD, *n* = 6).

To investigate the possible enhancement in pharmacokinetic behavior of FEX in blood, the plasma concentration versus time curve profiles of FEX after the oral administration of optimized FEX EIFV powder were compared to FEX tablet marketed products converted into powder. The results are illustrated in Figure 7. The pharmacokinetic parameters obtained from the study are listed in Table 6, the maximum concentration (C_max_) of optimized FEX EIFV formulation was 53.94 ± 6.09 µg/mL, compared with marketed product which was 37.28 ± 3.54 µg/mL. The maximum FEX plasma concentration was achieved in 1 h only compared to 3 h in the marketed product powder. The MRT was longer in the marketed product. Enhancing the absorption and increasing its rate is the aim of this study rather than prolongation of the effect of the drug, this was achieved in rabbits’ plasma data and can be related to the presence of span 40 as well as Capryol 90. The absorption half-life time of the optimized EIFV formulation was 1.75 ± 0.41 h and for the marketed drug is 2.39 ± 0.36 h Data also showed an increase in the AUC in the optimized formulation compared to the marketed product indicating higher oral bioavailability. The AUC_0–8h_ obtained from optimized formulation was found to be 212.22 ± 8.77 µg.h/mL, compared to 177.89 ± 8.16 µg.h/mL for the marketed product group. The absolute bioavailability of FEX was previously determined and found to be 35% [48]. The relative bioavailability of the optimized formulation was about 125% compared to the marketed product powder. Previous studies achieved high improved bioavailability and increase in the C_max_, but the t_max_ was relatively close to the marketed product [16]. In the current study, high decrease in t_max_ was found in rabbits administered the optimized formulation compared to the marketed product in its powdered form. The rapid absorption of FEX due to the presence of Capryol 90 was proved in different studies. Hu et al. studied the effect of the presence of Capryol 90 in microemulsion on the oral absorption of Fenofibrate in dogs, they found that Capryol 90 microemuslion achieved higher bioavailability of Fenofibrate compared to the marketed product and the microemuslion contains Labrafil M 1944 CS [49]. Kang et al. achieved 159% higher relative bioavailability of Simvastatin after oral administration over the marketed product by formulation of the drug into self-microemulsifying drug delivery system containing Capryol 90 as an oil [50]. The higher C_max_ obtained by the inclusion of Capryol 90 might be due to the role of Capryol 90 as an absorption enhancer which act as p-glycoprotein and/or CYP450 enzymes inhibitors decreasing intestinal efflux and drug biotransformation [13]. For the FEX being a substrate of p-glycoprotein which result in its low permeability [15], the inhibitory action of Capryol 90 to p-glycoprotein enhances the permeability of the FEX which resulted in an increase in its plasma concentration after oral administration of the optimized formulation.

### 3.6. Pharmacological Evaluation of the FEX in the Optimized Formula

#### Effect of FEX EIFV Optimized Powder on Compound 48/80-Induced Systemic Anaphylaxis-Like Reactions in Mice and Histamine Level in Plasma

In order to assess the pharmacological action of the FEX when prepared in EIFV, a systemic anaphylaxis-like reaction was induced in in vivo mice model using the mast cell degranulator compound 48/80. The morality percent among the mice was recorded and the histamine level was measured in plasma of the mice and their values are shown in Table 7. As seen from the results, 100% mortality rate was seen in the control group who administered 8 g/kg compound 48/80 by i.p. route. The poor oral bioavailability of FEX due to slow incomplete absorption resulted in high mortality rate although FEX is an effective antihistaminic agent. Both Telfast^®^ powdered tablet and the optimized EIFV powder showed higher efficiency in preventing the mortality due to anaphylactic shock. The optimized FEX EIFV inhibited the histamine release by 31.3% compared to only 21.4% inhibition in the marketed product powder group. While the poorly absorbed FEX powder achieved only 7.9% inhibition in the plasma histamine level after the administration of compound 48/80 in the fifth group. The formulation of FEX in EIFV powder with Capryol 90 resulted in high absorption and rapid onset. This resulted in a decrease in histamine level and increased survival percentage among the mice.

## 4. Conclusions

A novel provesicular system was developed and optimized to enhance the absorption of FEX by the addition of absorption enhancer to improve its bioavailability. The optimized free-flowing EIFV powder formulated contains span 40 as surfactant and Capryol 90 and the surfactant to Chol ratio is 1.268 and spray-dried lactose as a carrier. This formulation showed high entrapment efficiency and rapid in vitro release after 3 h. Central composite design was successfully applied in optimizing an HPLC assay method for Fexofenadine HCl in rabbit plasma. The study of response surface curves helped in better understanding the factors effect on method behavior and paved the way to obtain the optimum analysis conditions. Furthermore, the consideration of factor interactions and quadratic effects helped concluding a more reliable and robust HPLC method. The pharmacokinetic study on the optimized formulation in rabbits’ plasma showed an increase by 125% in the relative bioavailability compared to powdered Telfast^®^ crushed tablets. The prepared optimized formulation showed improved antihistaminic effect in Compound 48/80-induced systemic anaphylaxis-like reactions in mice by significantly decreasing the mortality rate and histamine level in plasma. Overall, the findings indicate that the optimized novel FEX EIFV is a promising solid nanocarrier for enhancing the oral absorption of FEX. 

## Figures and Tables

**Table 1:** The composition of the prepared Fexofenadine (FEX) enhanced in situ forming vesicles (EIFV) powder formulations.

Form.	X_1_: Surfactant Type	X_2_: Enhancer Type	X_3_: Surfactant: Cholesterol Ratio	Surfactant Weight (mg)	Enhancer Weight (mg)	Cholesterol Weight (mg)
F1	Span 40	None	1	500	0	500
F2	Span 40	None	2	666.66	0	333.33
F3	Span 60	None	1	500	0	500
F4	Span 60	None	2	666.66	0	333.33
F5	Span 40	Maisine CC	1	333.33	333.33	333.33
F6	Span 40	Maisine CC	2	500	250	250
F7	Span 60	Maisine CC	1	333.33	333.33	333.33
F8	Span 60	Maisine CC	2	500	250	250
F9	Span 40	Capryol 90	1	333.33	333.33	333.33
F10	Span 40	Capryol 90	2	500	250	250
F11	Span 60	Capryol 90	1	333.33	333.33	333.33
F12	Span 60	Capryol 90	2	500	250	250
F13	Span 40	Labrafil M 1944	1	333.33	333.33	333.33
F14	Span 40	Labrafil M 1944	2	500	250	250
F15	Span 60	Labrafil M 1944	1	333.33	333.33	333.33
F16	Span 60	Labrafil M 1944	2	500	250	250

Each formulation contains Fexofenadine HCl amount equals 120 mg. The ratios of surfactant to cholesterol used are 2:1 and 1:1 respectively. Since the Cholesterol ratio is always one, so the levels of the variable were written as 1 and 2. The ratio of enhancer: cholesterol is fixed to 1:1 in all formulation-containing enhancers. The Carrier spray-dried lactose amount is 1 g in all formulations (1:1 to the total weight of surfactant, enhancer and cholesterol).

**Table 2:** Particle size (PS), polydispersity index (PDI), zeta potential (ZP), entrapment efficiency (EE), and cumulative 3 h release (Q3h) of fexofenadine from the prepared fexofenadine HCl formulations.

Formula	PS (nm)	PDI	ZP (mV)	EE (%)	Q3h (%)
**F1**	276.0 ± 5.279	0.380 ± 0.002	−28.0 ± 0.208	55.44 ± 1.25	59.55 ± 2.15
**F2**	249.9 ± 2.261	0.357 ± 0.010	−24.3 ± 1.550	60.11 ± 0.92	66.14 ± 3.24
**F3**	282.9 ± 4.912	0.389 ± 0.019	−28.6 ± 0.902	71.63 ± 2.01	57.36 ± 4.20
**F4**	272.8 ± 2.261	0.306 ± 0.017	−23.5 ± 1.330	61.37 ± 0.55	63.53 ± 3.85
**F5**	262.4 ± 3.073	0.335 ± 0.039	−32.9 ± 1.250	56.44 ± 0.95	62.44 ± 1.11
**F6**	252.1 ± 5.382	0.267 ± 0.021	−30.6 ± 0.954	41.32 ± 1.13	79.65 ± 6.45
**F7**	304.6 ± 3.213	0.360 ± 0.029	−32.3 ± 0.755	44.33 ± 2.15	88.19 ± 3.90
**F8**	323.9 ± 3.109	0.378 ± 0.018	−32.6 ± 1.280	71.44 ± 1.55	76.14 ± 6.54
**F9**	237.3 ± 1.943	0.313 ± 0.005	−30.9 ± 1.440	59.67 ± 2.57	55.68 ± 1.25
**F10**	218.1 ± 4.729	0.281 ± 0.036	−29.6 ± 0.651	62.56 ± 0.63	81.23 ± 2.25
**F11**	269.6 ± 5.957	0.323 ± 0.018	−31.7 ± 0.751	55.66 ± 2.78	73.23 ± 3.40
**F12**	301.5 ± 2.572	0.323 ± 0.030	−30.2 ± 0.416	77.60 ± 3.21	72.13 ± 1.37
**F13**	235.1 ± 2.325	0.317 ± 0.037	−34.0 ± 1.140	44.43 ± 1.78	81.52 ± 1.98
**F14**	251.5 ± 4.852	0.346 ± 0.016	−32.4 ± 0.404	33.34 ± 0.99	73.21 ± 3.14
**F15**	269.6 ± 8.517	0.358 ± 0.045	−36.2 ± 0.874	45.82 ± 0.82	86.52 ± 1.21
**F16**	288.5 ± 1.457	0.384 ± 0.009	−33.7 ± 1.360	59.52 ± 3.15	69.33 ± 1.65

**Table 3:** The optimized variables, the predicted, and the observed responses of the optimal formulation.

**Variable**	**X_1_:Surfactant Type**	**X_2_:Enhancer Type**		**X_3_:Surfactant:Chol Ratio**
**Selected**	Span 40	Capryol 90		1.268
**Responses**	**Y_1_:PS (nm)**	**Y_2_:ZP (mV)**	**Y_3_:EE (%)**	**Y_4_:Q3h (%)**
**Predicted**	235.3	ȡ30.7	75.0	69.72
**Observed**	202.6 ± 3.90	−31.6 ± 0.92	73.65 ± 1.68	71.5 ± 2.65

(PS) particle size, (ZP) zeta potential, (EE) is the entrapment efficiency % of FEX in the vesicles, and (Q3h) is the cumulative 3 h release of FEX from the optimized EIFV formulation.

**Table 4:** Experimental domain of two-level CCD of HPLC assay optimization for three factors and measured responses.

Std.	Run	Factors Levels			Responses		
		pH	Column Temperature (°C)	Flow Rate (mL/min)	Rt FEX	Rt TNZ	Peak Area FEX
4	1	2.50	30.00	0.80	3.87	1.70	426,304
16	2	3.50	30.00	0.90	3.75	1.68	411,926
12	3	3.00	35.00	0.90	3.62	1.60	455,576
13	4	2.50	40.00	0.90	3.55	1.58	454,224
3	5	3.50	40.00	0.90	3.65	1.61	446,531
5	6	3.00	26.95	1.00	3.62	1.62	437,479
1	7	2.16	35.00	1.00	3.91	1.66	458,515
15	8	3.00	35.00	1.00	3.75	1.69	458,882
8	9	3.00	35.00	1.00	3.76	1.70	459,250
6	10	3.00	35.00	1.00	3.74	1.70	458,552
17	11	3.00	35.00	1.00	3.74	1.70	458,735
19	12	3.00	35.00	1.00	3.74	1.69	458,816
9	13	3.00	35.00	1.00	3.74	1.69	458,794
20	14	3.00	35.00	1.00	3.74	1.69	459,213
14	15	3.84	35.00	1.00	3.98	1.91	443,894
7	16	2.50	30.00	1.10	4.00	1.77	426,620
10	17	3.50	30.00	1.10	4.07	1.87	407,547
11	18	2.50	40.00	1.10	3.76	1.66	448,713
18	19	3.00	43.41	1.10	3.74	1.66	461,233
2	20	3.50	40.00	1.20	3.87	1.81	437,156

**Table 5:** Micromeritics of the prepared FEX EIFV.

Formula	Angle of Repose (θ)	Carr’s Index	Hausner Ratio
**F1**	28.15 ± 1.2	16.45 ± 1.01	1.197 ± 0.04
**F2**	23.45 ± 0.9	14.70 ± 0.72	1.172 ± 0.03
**F3**	25.89 ± 2.3	15.77 ± 0.92	1.187 ± 0.05
**F4**	26.17 ± 1.1	15.62 ± 0.85	1.185 ± 0.03
**F5**	41.08 ± 2.5	24.99 ± 0.66	1.333 ± 0.03
**F6**	38.19 ±2.8	22.53 ± 1.33	1.291 ± 0.06
**F7**	38.16 ± 2.1	23.00 ± 1.10	1.299 ± 0.05
**F8**	40.15 ± 2.5	25.36 ± 0.90	1.340 ± 0.04
**F9**	28.41 ± 1.6	16.51 ± 1.05	1.198 ± 0.03
**F10**	27.56 ± 1.2	17.17 ± 1.11	1.207± 0.04
**F11**	30.05 ± 2.8	17.50 ± 1.23	1.212 ± 0.04
**F12**	28.48 ± 0.9	16.11 ± 0.92	1.192 ± 0.03
**F13**	31.54 ± 1.7	18.84 ± 1.38	1.232 ± 0.06
**F14**	30.78 ± 2.2	16.24 ± 1.21	1.194 ± 0.04
**F15**	28.88 ± 1.9	18.51 ± 1.22	1.227 ± 0.05
**F16**	29.58 ± 2.3	17.65 ± 1.25	1.214 ± 0.05

**Table 6:** Pharmacokinetic data from the curve fitting of in vivo rabbit plasma data after administration of the optimized formulation and the marketed products (*n* = 6) with SD.

	t_max_ (h)	t_1/2_ (h)	C_max_ (μg/mL)	AUC_(0-8)_ (μg.h/mL)	MRT (h)
**Optimized FEX EIFV**	1.00 *	1.75 * ± 0.41	53.94 * ± 6.09	212.22 * ± 8.77	3.25 * ± 0.33
**Marketed product**	3.00	2.39 ± 0.36	37.28 ± 3.54	177.89 ± 8.16	3.89 ± 0.24

Abbreviations: (AUC) the area under the curve, (MRT) the mean residence time, (SD) the standard deviation. * Significant difference (*p* < 0.05).

**Table 7:** Effect of pure drug, marketed product and optimized formulation on the compound 48/80 induced systemic anaphylactic reaction in mice (*n* = 8) with SD.

	Treatment	Compound 48/80 (8 mg/kg)	Mortality	Histamine Concentration (ng/mL)
**Group 1**	**None (PBS)**	-	0%	111.7 ± 7.9
**Group 2**	**None (PBS)**	+	100%	256.8 ± 13.5
**Group 3**	**FEX powder**	+	62.5% *	236.4 ± 12.9
**Group 4**	**Marketed product powder**	+	12.5% *	201.8 ± 9.2
**Group 5**	**Optimized FEX EIFV powder**	+	12.5% *	176.3 ± 11.8

Mortality (%) within 1 h following the i.p injection of compound 48/80 was represented as no. of dead miceX100/total no. of experimental mice. * Significant difference from the positive control group (*p* < 0.05).

## References

[1] Borges A.F., Silva C., Coelho J.F., Simões S. (2015). Oral films: Current status and future perspectives: I—Galenical development and quality attributes. J. Control. Release.

[2] Wang Y., Zhao Y., Cui Y., Zhao Q., Zhang Q., Musetti S., Kinghorn K.A., Wang S. (2018). Overcoming multiple gastrointestinal barriers by bilayer modified hollow mesoporous silica nanocarriers. Acta Biomater..

[3] Sammour O.A., Hammad M.A., Zidan A.S., Mowafy A.G. (2011). QbD approach of rapid disintegrating tablets incorporating indomethacin solid dispersion. Pharm. Dev. Technol..

[4] Massart D.L., Vandeginste B.G.M., Buydens L.M.C., Jong S.D., Smeyers-Verbeke J. (1997). Handbook of Chemometrics and Qualimetrics: Part A.

[5] Dejaegher B., Heyden Y.V. (2011). Experimental designs and their recent advances in set-up, data interpretation, and analytical applications. J. Pharm. Biomed. Anal..

[6] Hobson J.J., Edwards S., Slater R.A., Martin P., Owen A., Rannard S.P. (2018). Branched copolymer-stabilised nanoemulsions as new candidate oral drug delivery systems. RSC Adv..

[7] Bayindir Z.S., Yuksel N. (2015). Provesicles as novel drug delivery systems. Curr. Pharm. Biotechnol..

[8] Mokhtar M., Sammour O.A., Hammad M.A., Megrab N.A. (2008). Effect of some formulation parameters on flurbiprofen encapsulation and release rates of niosomes prepared from proniosomes. Int. J. Pharm..

[9] Yasam V.R., Jakki S.L., Natarajan J., Kuppusamy G. (2014). A review on novel vesicular drug delivery: Proniosomes. Drug Deliv..

[10] Bandyopadhyay S., Katare O., Singh B. (2012). Optimized self nano-emulsifying systems of ezetimibe with enhanced bioavailability potential using long chain and medium chain triglycerides. Colloids Surf. B Biointerfaces.

[11] Holm R., Porter C.J., Edwards G.A., Müllertz A., Kristensen H.G., Charman W.N. (2003). Examination of oral absorption and lymphatic transport of halofantrine in a triple-cannulated canine model after administration in self-microemulsifying drug delivery systems (SMEDDS) containing structured triglycerides. Eur. J. Pharm. Sci..

[12] Han M., Fu S., Gao J.-Q., Fang X.-L. (2009). Evaluation of intestinal absorption of ginsenoside Rg1 incorporated in microemulison using parallel artificial membrane permeability assay. Biol. Pharm. Bull..

[13] Basalious E.B., Shawky N., Badr-Eldin S.M. (2010). SNEDDS containing bioenhancers for improvement of dissolution and oral absorption of lacidipine. I: Development and optimization. Int. J. Pharm..

[14] Eedara B.B., Veerareddy P.R., Jukanti R., Bandari S. (2014). Improved oral bioavailability of fexofenadine hydrochloride using lipid surfactants: Ex vivo, in situ and in vivo studies. Drug Dev. Ind. Pharm..

[15] Drescher S., Schaeffeler E., Hitzl M., Hofmann U., Schwab M., Brinkmann U., Eichelbaum M., Fromm M.F. (2002). MDR1 gene polymorphisms and disposition of the P-glycoprotein substrate fexofenadine. Br. J. Clin. Pharmacol..

[16] Gundogdu E., Alvarez I.G., Karasulu E. (2011). Improvement of effect of water-in-oil microemulsion as an oral delivery system for fexofenadine: In vitro and in vivo studies. Int. J. Nanomed..

[17] Bousquet J., Khaltaev N., Cruz A.A., Denburg J., Fokkens W., Togias A., Zuberbier T., Baena-Cagnani C., Canonica G., Van Weel C. (2008). Allergic rhinitis and its impact on asthma (ARIA) 2008. Allergy.

[18] Nasr A., Qushawy M., Swidan S. (2018). Spray Dried Lactose Based Proniosomes as Stable Provesicular Drug Delivery Carriers: Screening, Formulation, and Physicochemical Characterization. Int. J. Appl. Pharm..

[19] Kumar G.P., Rajeshwarrao P. (2011). Nonionic surfactant vesicular systems for effective drug delivery—An overview. Acta Pharm. Sin. B.

[20] Blazek-Welsh A.I., Rhodes D.G. (2001). Maltodextrin-based proniosomes. Aaps Pharmsci.

[21] Al-Hashemi H.M.B., Al-Amoudi O.S.B. (2018). A review on the angle of repose of granular materials. Powder Technol..

[22] Jaimini M., Rana A., Tanwar Y. (2007). Formulation and evaluation of famotidine floating tablets. Curr. Drug Deliv..

[23] Veerareddy P.R., Bobbala S.K.R. (2013). Enhanced oral bioavailability of isradipine via proniosomal systems. Drug Dev. Ind. Pharm..

[24] Food and Drug Administration of the United States (US-FDA), U.S. Department of Health and Human Services (DHHS), Center for Drug Evaluation and Research (CDER), Center for Veterinary Medicine (CVM) (2018). Bioanalytical Method Validation. Guidance for Industry.

[25] Choi Y.H., Chai O.H., Han E.-H., Choi S.-Y., Kim H.T., Song C.H. (2010). Lipoic acid suppresses compound 48/80-induced anaphylaxis-like reaction. Anat. Cell Biol..

[26] Shin T., Park J., Kim H. (1999). Effect of Cryptotympana atrata extract on compound 48/80-induced anaphylactic reactions. J. Ethnopharmacol..

[27] Shore P.A. (1959). A method for the fluorometric assay of histamine in tissues. J. Pharmacol. Exp. Ther..

[28] Uchegbu I.F., Vyas S.P. (1998). Non-ionic surfactant based vesicles (niosomes) in drug delivery. Int. J. Pharm..

[29] Balakrishnan P., Shanmugam S., Lee W.S., Lee W.M., Kim J.O., Oh D.H., Kim D.-D., Kim J.S., Yoo B.K., Choi H.-G. (2009). Formulation and in vitro assessment of minoxidil niosomes for enhanced skin delivery. Int. J. Pharm..

[30] Izham M., Nadiah M., Hussin Y., Aziz M.N.M., Yeap S.K., Rahman H.S., Masarudin M.J., Mohamad N.E., Abdullah R., Alitheen N.B. (2019). Preparation and Characterization of Self Nano-Emulsifying Drug Delivery System Loaded with Citraland Its Antiproliferative Effect on Colorectal Cells In Vitro. Nanomaterials.

[31] Parmar N., Singla N., Amin S., Kohli K. (2011). Study of cosurfactant effect on nanoemulsifying area and development of lercanidipine loaded (SNEDDS) self nanoemulsifying drug delivery system. Colloids Surf. B Biointerfaces.

[32] Kamboj S., Saini V., Bala S. (2014). Formulation and characterization of drug loaded nonionic surfactant vesicles (niosomes) for oral bioavailability enhancement. Sci. World J..

[33] Nasr M., Mansour S., Mortada N.D., Elshamy A. (2008). Vesicular aceclofenac systems: A comparative study between liposomes and niosomes. J. Microencapsul..

[34] El-Alim S.A., Kassem A., Basha M. (2014). Proniosomes as a novel drug carrier system for buccal delivery of benzocaine. J. Drug Deliv. Sci. Technol..

[35] Basiri L., Rajabzadeh G., Bostan A. (2017). α-Tocopherol-loaded niosome prepared by heating method and its release behavior. Food Chem..

[36] Liu T., Guo R., Hua W., Qiu J. (2007). Structure behaviors of hemoglobin in PEG 6000/Tween 80/Span 80/H2O niosome system. Colloids Surf. A Physicochem. Eng. Asp..

[37] Ahmed A., Ghourab M., Shedid S., Qushawy M. (2013). Optimization of piroxicam niosomes using central composite design. Int. J. Pharm. Pharm. Sci..

[38] Yoshioka T., Sternberg B., Florence A.T. (1994). Preparation and properties of vesicles (niosomes) of sorbitan monoesters (Span 20, 40, 60 and 80) and a sorbitan triester (Span 85). Int. J. Pharm..

[39] Nagarsenker M., Londhe V. (2003). Preparation and evaluation of a liposomal formulation of sodium cromoglicate. Int. J. Pharm..

[40] Fatouros D., Hatzidimitriou K., Antimisiaris S. (2001). Liposomes encapsulating prednisolone and prednisolone–cyclodextrin complexes: Comparison of membrane integrity and drug release. Eur. J. Pharm. Sci..

[41] Attia I.A., El-Gizawy S.A., Fouda M.A., Donia A.M. (2007). Influence of a niosomal formulation on the oral bioavailability of acyclovir in rabbits. AAPS PharmSciTech.

[42] Al-mahallawi A.M., Khowessah O.M., Shoukri R.A. (2014). Nano-transfersomal ciprofloxacin loaded vesicles for non-invasive trans-tympanic ototopical delivery: In-vitro optimization, ex-vivo permeation studies, and in-vivo assessment. Int. J. Pharm..

[43] Solanki A.B., Parikh J.R., Parikh R.H. (2007). Formulation and optimization of piroxicam proniosomes by 3-factor, 3-level Box-Behnken design. AAPS PharmSciTech.

[44] Basha M., Abd El-Alim S.H., Shamma R.N., Awad G.E. (2013). Design and optimization of surfactant-based nanovesicles for ocular delivery of Clotrimazole. J. Liposome Res..

[45] Kumar L., Alam M.S., Meena C.L., Jain R., Bansal A.K. (2009). Fexofenadine hydrochloride. Profiles of Drug Substances, Excipients and Related Methodology.

[46] Gurrapu A., Jukanti R., Bobbala S.R., Kanuganti S., Jeevana J.B. (2012). Improved oral delivery of valsartan from maltodextrin based proniosome powders. Adv. Powder Technol..

[47] Aburahma M.H., Abdelbary G.A. (2012). Novel diphenyl dimethyl bicarboxylate provesicular powders with enhanced hepatocurative activity: Preparation, optimization, in vitro/in vivo evaluation. Int. J. Pharm..

[48] Chen C. (2007). Some pharmacokinetic aspects of the lipophilic terfenadine and zwitterionic fexofenadine in humans. Drugs R D.

[49] Hu L., Wu H., Niu F., Yan C., Yang X., Jia Y. (2011). Design of fenofibrate microemulsion for improved bioavailability. Int. J. Pharm..

[50] Kang B.K., Lee J.S., Chon S.K., Jeong S.Y., Yuk S.H., Khang G., Lee H.B., Cho S.H. (2004). Development of self-microemulsifying drug delivery systems (SMEDDS) for oral bioavailability enhancement of simvastatin in beagle dogs. Int. J. Pharm..

